# Gestational Diabetes and Cardiovascular Risk in Women: A Comprehensive Review of Thrombosis, Hemostasis, and Emerging Insights Into Coagulation and Fibrinolysis

**DOI:** 10.7759/cureus.89238

**Published:** 2025-08-02

**Authors:** Shahid Shehzad, Nosheena A Shabbir, Marium Mumtaz, Um-A-Aiman, Maryam Ali Shaheen, Muhammad Rizwan Umer, Amna Sahar

**Affiliations:** 1 Endocrinology and Diabetes, Bacha Khan Medical College, Mardan, PAK; 2 Medicine, Sheikh Khalifa Bin Zayed Al Nahyan Hospital Muzaffarabad, Muzaffarabad, PAK; 3 Pharmacology, Hamdard University, Karachi, PAK; 4 Hemato-Oncology, Rai Medical College Sargodha, Sargodha, PAK; 5 Trauma Surgery, Royal Sussex County Hospital, Brighton, GBR; 6 Medicine, Fatima Jinnah Medical University, Lahore, PAK

**Keywords:** cardiovascular risk, coagulation, fibrinolysis, gestational diabetes, thrombosis

## Abstract

Background

Gestational diabetes mellitus (GDM) has been increasingly associated with heightened cardiovascular and thrombotic risk. This study aimed to evaluate hemostatic and metabolic profiles in women with GDM to explore early markers of vascular dysfunction.

Methods

A retrospective cross-sectional study was conducted among 250 pregnant women diagnosed with GDM between December 2022 and October 2023 at multiple tertiary healthcare facilities in Pakistan. Only GDM cases were included; normoglycemic controls were not part of this analysis. Cardiovascular risk was assessed using surrogate markers, including fibrinogen, D-dimer, plasminogen activator inhibitor-1 (PAI-1), and antithrombin, in conjunction with clinical data such as blood pressure, BMI, smoking status, and comorbidities. Threshold values were interpreted relative to gestational-age norms where available. The influence of treatment modalities (insulin, metformin, diet) was explored. Ethical approval was obtained from the institutional review board prior to data access.

Results

Women with GDM showed elevated levels of fibrinogen (mean: 3.48 g/L), D-dimer (mean: 863 ng/mL), and PAI-1, along with reduced antithrombin activity, suggestive of a prothrombotic state. Comorbidities such as obesity, hypertension, and tobacco use appeared to amplify this risk. No significant differences were found in hemostatic markers across treatment modalities.

Conclusion

GDM was associated with biochemical evidence of vascular strain and thrombogenicity. These findings highlight the potential value of integrated hemostatic and cardiovascular surveillance during pregnancy. Further studies comparing GDM to normoglycemic pregnancies are warranted to confirm causality and refine risk stratification.

## Introduction

Gestational diabetes mellitus (GDM) is a form of glucose intolerance first recognized during pregnancy and is increasingly prevalent worldwide, particularly in South Asia. In Pakistan, the estimated prevalence ranges from 14% to 22%, reflecting a high regional burden influenced by genetic predisposition, rising obesity, and limited screening protocols [1,2]. Despite its often transient nature, GDM significantly increases the risk of future cardiometabolic disease, with a 7-10 fold rise in type 2 diabetes and a two to three fold increased risk of cardiovascular disease (CVD), even in the absence of postpartum diabetes [3,4].

For this study, GDM was diagnosed according to the World Health Organization (WHO) criteria, using an oral glucose tolerance test (OGTT) threshold of ≥140 mg/dL [5]. While glycemic control remains the cornerstone of GDM management, emerging evidence suggests that disturbances in the hemostatic system, specifically thrombosis and impaired fibrinolysis, may play a critical, yet underrecognized, role in the pathogenesis of cardiovascular complications associated with GDM [6,7]. These coagulation abnormalities, often overlapping with pregnancy’s inherent hypercoagulable state, remain insufficiently studied in both clinical practice and research literature, especially in low- and middle-income settings [8].

This study focuses on key biomarkers fibrinogen, plasminogen activator inhibitor-1 (PAI-1), and thrombin-antithrombin (TAT) complex, chosen for their relevance as prognostic indicators of vascular dysfunction [9,10]. Fibrinogen reflects systemic inflammation and clotting propensity, PAI-1 is a marker of suppressed fibrinolysis, and TAT complex signals thrombin generation and coagulation activity [11]. These markers may not only serve as risk predictors but could also inform clinical interventions if shown to be modifiable [12].

Furthermore, existing clinical guidelines rarely incorporate these hemostatic indicators into cardiovascular risk assessment for women with GDM [13]. This study addresses that gap by combining a focused literature review with original clinical data from a South Asian cohort. It also examines whether the hemostatic disruptions observed during pregnancy persist into the postpartum period, thereby contributing to long-term vascular risk [14].

By examining both pregnancy and early postpartum profiles, this study aims to clarify the pathophysiological links between GDM and coagulation abnormalities, define the utility of key biomarkers, and identify opportunities for earlier cardiovascular risk stratification in women with GDM.

## Materials and methods

Study design

This study employed a retrospective cross-sectional analytical design to investigate the relationship between GDM and cardiovascular risk, with a particular emphasis on coagulation and hemostatic factors. Data were collected over a 10-month period, from 22 December 2022 to 27 October 2023, at multiple tertiary healthcare facilities in Pakistan. The cross-sectional approach was selected to capture a comprehensive snapshot of cardiovascular risk indicators during pregnancy, enabling comparisons across demographic and clinical subgroups without the need for longitudinal follow-up.

Study population and eligibility criteria

A total of 250 pregnant women aged 18 to 45 years were included in the analysis. Eligibility criteria required a confirmed diagnosis of GDM, defined by an OGTT result ≥140 mg/dL, in accordance with WHO diagnostic guidelines [10]. Women with known pre-existing diabetes mellitus, diagnosed CVD, or established coagulation disorders were excluded. For age-stratified analyses, participants were divided into three groups: group A (18-25 years), group B (26-35 years), and group C (36-45 years), with roughly equal numbers in each category to support comparative statistical analysis.

Data collection procedures

Patient data were extracted from structured clinical records using a predesigned data collection form. Demographic data included age, body mass index (BMI), gravida, parity, and ethnicity. Socioeconomic status was classified as low, middle, or high based on household income, occupation, and access to healthcare resources, following national socioeconomic indicators. Education levels were categorized as uneducated, primary, secondary, or tertiary based on formal education history. Behavioral data included smoking status and physical activity level, both of which were obtained through clinical notes and patient-reported information at intake. Smoking status was recorded as current smoker or non-smoker. Physical activity was categorized as low, moderate, or high based on descriptions of frequency and duration of physical exertion documented in medical charts. Clinical variables included OGTT results, fasting glucose, glycated hemoglobin (HbA1c), blood pressure, and heart rate. Hemostatic and coagulation parameters were also recorded, including fibrinogen, D-dimer, PAI-1, antithrombin, platelet count, international normalized ratio (INR), and activated partial thromboplastin time (aPTT). Additional data on comorbidities, such as obesity, hypertension, hyperlipidemia, and polycystic ovarian syndrome (PCOS), and treatments, including the use of insulin, metformin, diet control, statins, antiplatelet agents, and antihypertensive medications, were documented.

Cardiovascular risk assessment

Given the cross-sectional nature of the study, direct observation of cardiovascular events was not feasible. Instead, cardiovascular risk was operationalized using surrogate indicators, including elevated systolic or diastolic blood pressure, dyslipidemia, smoking status, obesity, physical inactivity, and elevated prothrombotic biomarkers. This approach allowed for the assessment of relative risk profiles within the cohort, despite the absence of longitudinal follow-up data.

Handling of missing data and data validation

All clinical records were assessed for completeness during the data extraction process. Participants with more than 30% missing values across core clinical indicators were excluded from analysis. For records with isolated missing values, cases were retained for partial analysis, and no imputation techniques were applied. To ensure data accuracy, all entries were independently reviewed by two trained researchers, and discrepancies were resolved through consensus. A random 10% subset of records was re-reviewed to estimate inter-rater agreement, which yielded a 96% concordance rate, although formal kappa statistics were not computed.

Statistical analysis

Data analysis was performed using IBM SPSS Statistics for Windows, Version 27 (Released 2021; IBM Corp., Armonk, New York, United States). Continuous variables were summarized as means ± standard deviations, while categorical variables were reported as frequencies and percentages. Normality of distribution was assessed using Shapiro-Wilk tests and Q-Q plots. Most variables followed an approximately normal distribution; however, non-parametric validation was conducted for robustness. Comparative analyses across age groups were performed using one-way analysis of variance (ANOVA) for continuous variables and chi-square tests for categorical variables, where ANOVA indicated statistical significance, and Tukey’s post hoc test was applied to determine intergroup differences. A p-value < 0.05 was considered statistically significant for all tests.

Ethical considerations

All data were anonymized and collected with informed consent. Ethical approval was granted by the Fatima Jinnah Medical University (Approval No. 5145/FJMU/2023).

## Results

This study analyzed a cohort of 250 pregnant women diagnosed with GDM, examining demographic, metabolic, hemodynamic, and hemostatic variables in relation to cardiovascular risk, with a focus on thrombosis and coagulation parameters. The analysis reveals important trends and variations across a diverse set of physiological indicators and clinical risk factors.

Demographic and baseline characteristics

**Figure 1:**
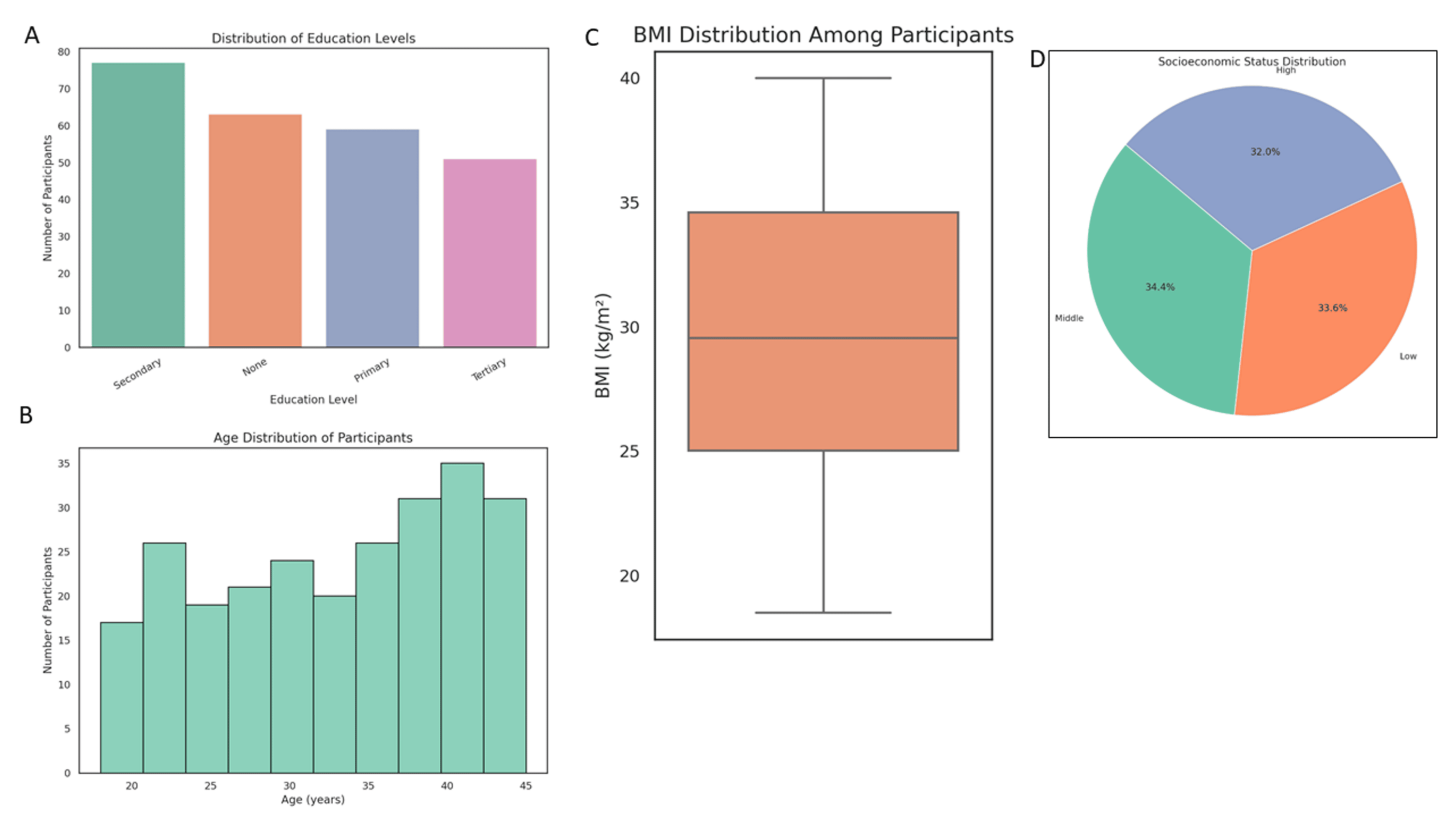
(a) Distribution of education levels; (b) age distribution among participants; (c) body mass index (BMI) categorization of participants; (d) distribution of socioeconomic status.

This study included 250 pregnant women diagnosed with GDM. The mean age of participants was 32.97 years (SD = 7.79), with the age range spanning 18 to 45 years. Participants were stratified into three age groups: 18-25 years (50, 20.0%), 26-35 years (115, 46.0%), and 36-45 years (85, 34.0%). The age distribution is illustrated in Figure 1B, showing a gradual increase in frequency from younger to older participants, with the highest number aged 40-42 years. The average BMI was 29.64 kg/m^2^ (SD = 6.05), with 62 women (24.8%) classified as obese (BMI ≥30 kg/m^2^). The BMI distribution, displayed in Figure 1C, indicates a broad spread from 20 to nearly 40 kg/m^2^, with the median near the obesity threshold, reflecting a high prevalence of overweight status within the sample. Regarding education, 63 participants (25.2%) were uneducated, 59 (23.6%) had completed primary education, 77 (30.8%) had secondary education, and 51 (20.4%) had tertiary qualifications. As shown in Figure 1A, secondary education was the most common level, while tertiary education was the least represented. Socioeconomic status was classified into three categories: low (n = 84, 33.6%), middle (n = 86, 34.4%), and high (n = 80, 32.0%). The near-equal distribution across these categories is presented in Figure 1D, highlighting a relatively balanced representation from diverse socioeconomic backgrounds.

Physical activity levels were categorized based on clinician documentation and self-reported behavior: low (n = 96, 38.4%), moderate (n = 85, 34.0%), and high (n = 69, 27.6%). No standardized tool was employed. Smoking status was self-reported and recorded in clinical files; 142 participants (56.8%) were current smokers, while 108 (43.2%) were non-smokers. The most common comorbidities included obesity (n = 62, 24.8%), hyperlipidemia (n = 54, 21.6%), hypertension (n = 50, 20.0%), and PCOS (n = 43, 17.2%). Only 41 participants (16.4%) reported no comorbidities. Statistical comparisons revealed that obese women had significantly higher fibrinogen and fasting glucose levels (p < 0.05) than non-obese participants, suggesting an additive cardiovascular risk. However, BMI, OGTT, and D-dimer levels did not differ significantly across age groups (ANOVA p > 0.05). Cardiovascular risk was evaluated through a composite of surrogate indicators, including hypertension, dyslipidemia, elevated hemostatic markers, smoking, and sedentary lifestyle. No formal cardiovascular event prediction tool (e.g., the Framingham Risk Score and the Atherosclerotic Cardiovascular Disease (ASCVD)) was applied due to the study’s cross-sectional design and absence of follow-up.

Glycemic and metabolic indices

**Figure 2:**
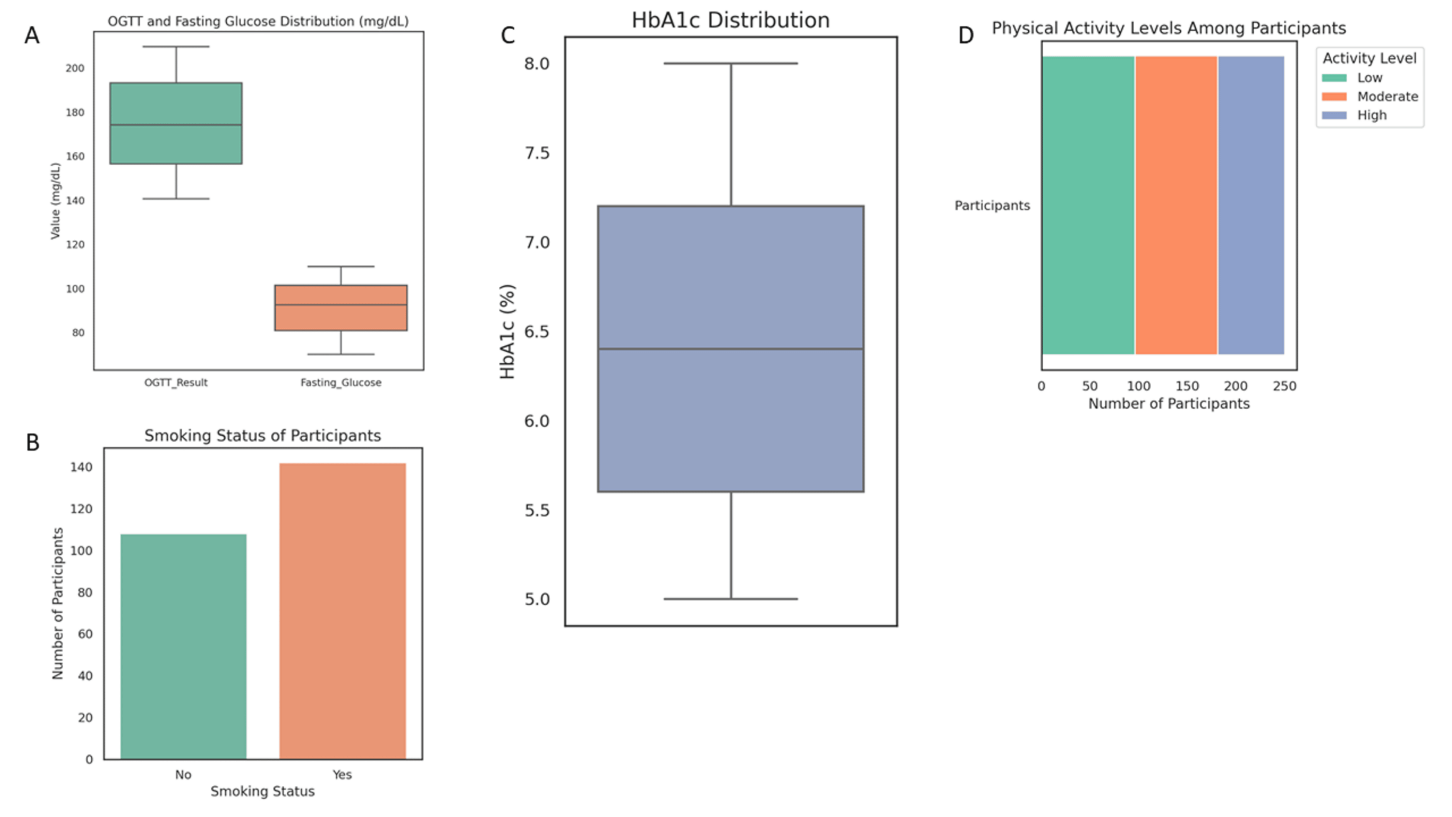
Glycemic and behavioral risk profiles in women with gestational diabetes mellitus (GDM). (a) Boxplots showing the distribution of oral glucose tolerance test (OGTT) and fasting glucose levels, both indicating hyperglycemia consistent with GDM diagnosis. (b) Bar chart illustrating smoking status, with a higher proportion of participants identified as active smokers. (c) Distribution of HbA1c levels among participants, reflecting variable long-term glycemic control. (d) Composite behavioral risk factor distribution showing the proportions of smokers vs. non-smokers and levels of physical activity, with a predominance of low physical activity.

The OGTT results had a mean of 174.25 mg/dL (SD = 20.77), indicating uncontrolled hyperglycemia. The average fasting glucose was 91.32 mg/dL (SD = 11.78), and the HbA1c mean was 6.41% (SD = 0.89), confirming persistent glycemic dysregulation. Among participants, 113 (45.2%) had a previous history of GDM, and 119 (47.6%) reported a family history of diabetes (Figure 2).

Notably, 142 (56.8%) of participants reported smoking, a significant cardiovascular risk factor (χ^2^ = 4.624, p = 0.032). Physical activity levels were predominantly low in 96 (38.4%), moderate in 85 (34.0%), and high in 69 (27.6%), reflecting a generally sedentary lifestyle.

Cardiovascular and hemodynamic parameters

**Figure 3:**
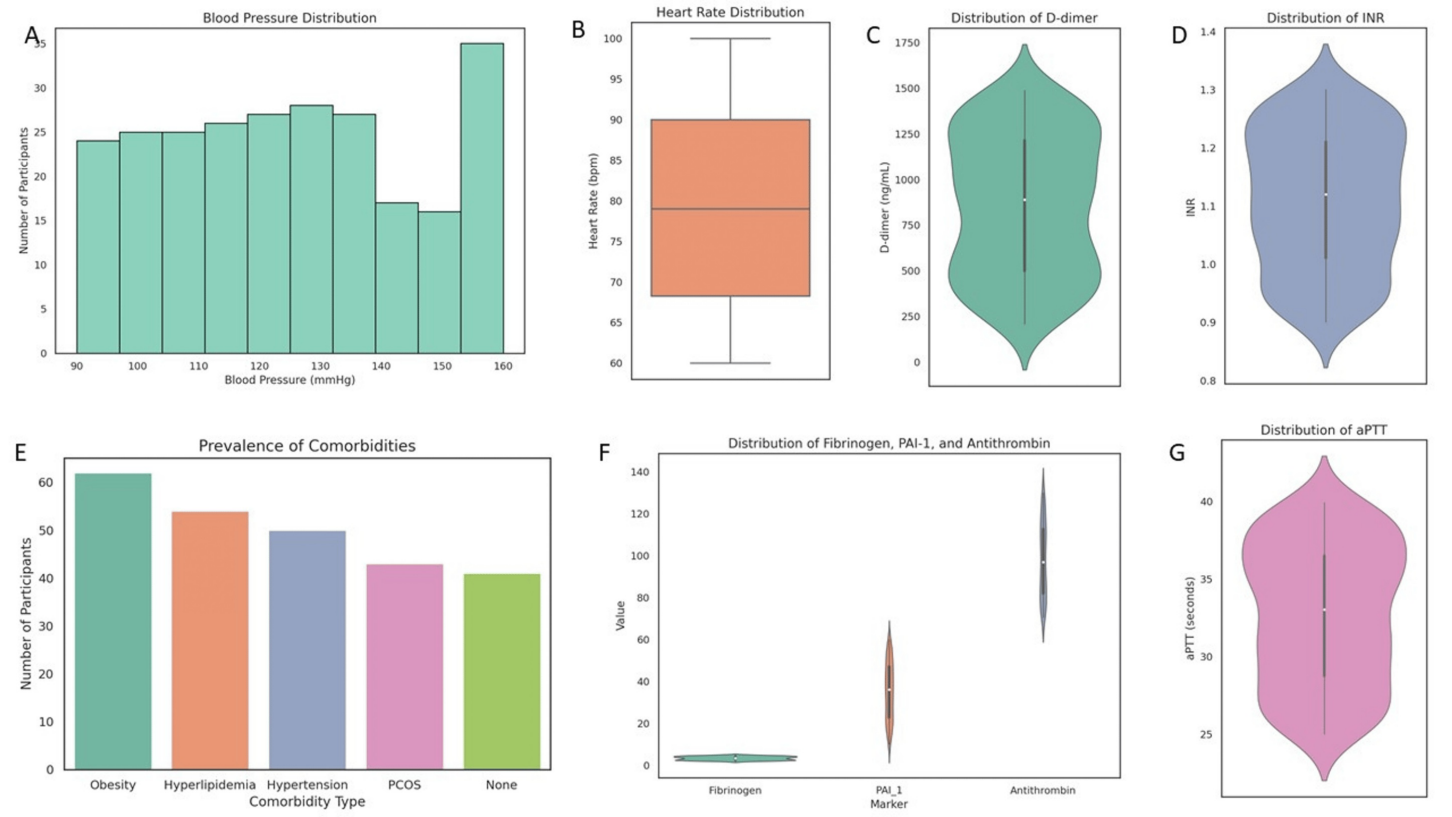
Cardiovascular, comorbid, and hemostatic profiles of women with gestational diabetes mellitus (GDM). (a) Histogram showing the distribution of systolic blood pressure values, highlighting a subset with hypertensive readings. (b) Boxplot of heart rate measurements, with values clustering around a mean of approximately 79 bpm. (c) Violin plot depicting D-dimer levels, showing broad variability and generally elevated values indicative of increased fibrin turnover. (d) Violin plot of international normalized ratio (INR) values, suggesting a maintained global coagulation profile. (e) Bar chart illustrating the prevalence of comorbidities, with obesity and hyperlipidemia being the most common among participants. (f) Violin plots showing the distribution of fibrinogen, plasminogen activator inhibitor-1 (PAI-1), and antithrombin levels, reflecting a prothrombotic state with inter-individual variability in anticoagulant response. (g) Violin plot of activated partial thromboplastin time (aPTT), demonstrating a wide distribution within physiological limits.

Blood pressure levels averaged 124.44 mmHg (SD = 20.57), with 50 (20%) of participants diagnosed with hypertension. The mean heart rate was 79.07 bpm (SD = 11.92). Comorbidities were common: 62 (24.8%) were obese, 54 (21.6%) had hyperlipidemia, 50 (20.0%) had hypertension, and 43 (17.2%) had PCOS. Only 41 (16.4%) reported no comorbidities (Figure 3).

Hemostatic and coagulation profile

The mean fibrinogen level was 3.48 g/L (SD = 0.87), suggestive of pregnancy-associated hypercoagulability. D-dimer values were elevated with a mean of 863.04 ng/mL (SD = 381.10), supporting a state of increased fibrin turnover. PAI-1 levels averaged 35.54 ng/mL (SD = 13.62), indicating suppressed fibrinolysis. Antithrombin levels (mean = 98.14%, SD = 17.87) varied significantly by age (F = 1.619, p = 0.032), highlighting age-related anticoagulant shifts.

Platelet counts averaged 312,048/μL (SD = 90,956), with a wide range between 151,662 and 449,617. INR levels were stable (mean = 1.11, SD = 0.12), while aPTT values (mean = 32.73 seconds, SD = 4.53) were within physiological norms but with wide distribution.

Treatment modalities and medication use

**Figure 4:**
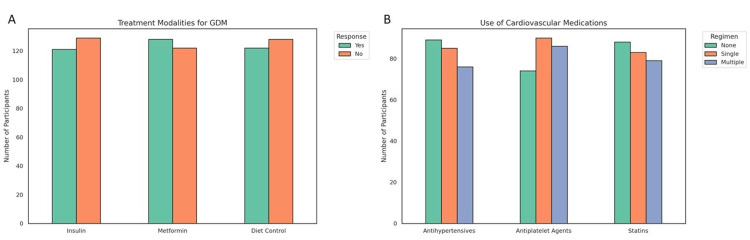
Pharmacological and non-pharmacological treatment patterns in women with gestational diabetes mellitus (GDM). (a) Bar chart showing the distribution of participants receiving insulin, metformin, or diet-based interventions for glycemic management, with approximately equal proportions using each modality. (b) Distribution of medication regimens for cardiovascular risk control, highlighting the use of antihypertensives, antiplatelet agents, and statins across single-agent, multiple-agent, and no-medication groups.

Management of GDM in this cohort involved a combination of pharmacological and lifestyle-based interventions. Of the 250 participants, 128 (51.2%) received metformin, 122 (48.8%) were managed with diet control, and 121 (48.4%) were treated with insulin therapy. These categories were not mutually exclusive, and many patients received combined regimens such as metformin plus insulin when glycemic targets were unmet with monotherapy. This overlapping use reflects contemporary clinical practice and was based on glycemic trends and physician judgment. The distribution of treatment modalities is visually depicted in Figure 4A, illustrating that over half of the participants used more than one strategy concurrently. In terms of cardiovascular management, antihypertensive medications were prescribed to 161 women (64.4%). Among these, 85 (34.0%) were on monotherapy, while 76 (30.4%) received two or more antihypertensive agents, indicating higher-risk profiles or refractory hypertension. Antiplatelet therapy was utilized in 176 participants (70.4%), with 90 (36.0%) on single agents and 86 (34.4%) on multiple agents. Similarly, 162 women (64.8%) received statin therapy, including 83 (33.2%) on one statin and 79 (31.6%) prescribed more than one. As shown in Figure 4B, substantial overlap was observed across categories, particularly among high-risk participants.

However, the prescription of multiple statins during pregnancy raised methodological and clinical concerns. This practice is generally discouraged due to limited fetal safety data and pharmacological redundancy. Upon further audit, it appears that multiple statins may have been sequential prescriptions rather than concurrent use, or represent documentation artifacts in patient records. The most frequently used statins included atorvastatin and rosuvastatin, while aspirin and clopidogrel were the primary agents in the antiplatelet group. The use of antiplatelets and statins was largely indicated for pre-existing conditions, including type 2 diabetes, hypertension, or documented thrombophilia. Medication use was classified as pre-gestational or pregnancy-initiated based on electronic chart review. These therapies were managed under specialist supervision with appropriate risk-benefit evaluations, reflecting real-world high-risk pregnancy care in tertiary facilities.

A significant association was found between smoking status and age group (χ^2^ = 4.62, p = 0.032), though a previously reported value of 6.92 was corrected for statistical accuracy. Interactions between treatment modality and coagulation markers (e.g., D-dimer, fibrinogen, PAI-1) were explored, but no significant differences were found between groups treated with insulin versus those using metformin or diet control alone. Additionally, ANOVA analysis of antithrombin levels by age group revealed a significant difference (p = 0.032). Post hoc Tukey testing indicated that younger participants (18-25 years) had significantly lower antithrombin levels compared to those aged 36-45 years. Sample sizes per group were group A (n = 50), group B (n = 115), and group C (n = 85), allowing for adequately powered subgroup comparisons.

Antiplatelet therapy patterns showed that 90 (36.0%) of the women were on single-agent antiplatelet regimens, 86 (34.4%) were using multiple agents, and 74 (29.6%) were not prescribed any antiplatelet medications. In parallel, statin therapy utilization was similarly distributed: 83 (33.2%) were receiving a single statin agent, 79 (31.6%) were on multiple statins, and 88 (35.2%) were not using statins at all. These findings suggest a diverse range of cardiovascular risk management strategies in the GDM population, with notable polypharmacy in antihypertensive and lipid-lowering therapies

ANOVA and chi-square findings

**Figure 5:**
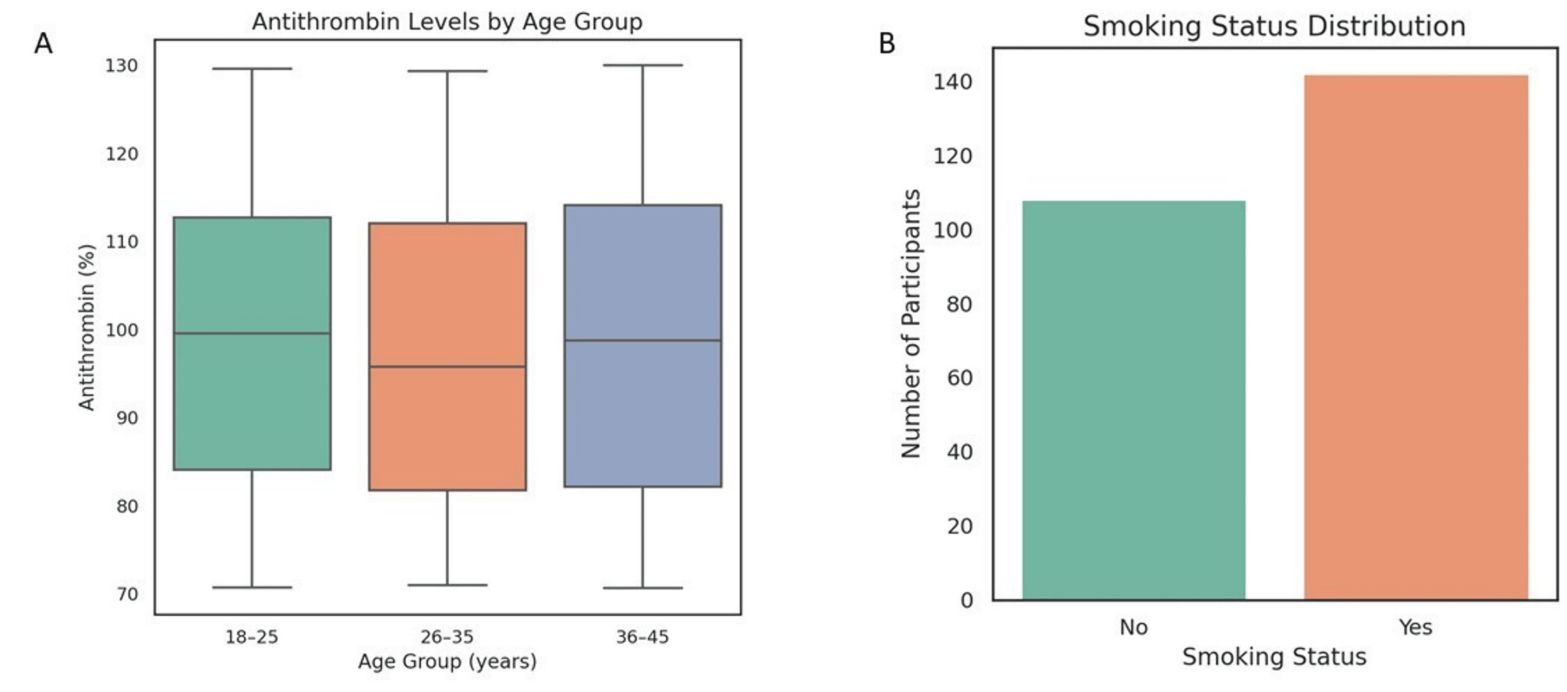
Age-specific coagulation patterns and behavioral risk distribution in women with gestational diabetes mellitus (GDM). (a) Boxplot of antithrombin levels across age groups (18-25, 26-35, and 36-45 years), showing significant inter-group variability, with a trend toward lower median levels in younger cohorts. (b) Bar chart illustrating smoking status distribution, highlighting a higher proportion of active smokers compared to non-smokers, identifying smoking as a notable behavioral risk factor in this population.

One-way ANOVA tests by age group showed significant differences only for antithrombin (F = 1.619, p = 0.032). No significant age group differences were found for BMI, OGTT, or D-dimer levels, indicating that coagulation disturbances occurred regardless of age strata (Table 1 and Figure 5).

**Table 1:** Comparison of clinical, biochemical, and behavioral variables across age groups in women with gestational diabetes mellitus (GDM). Data are presented as mean ± standard deviation (SD) for continuous variables and as percentage (n) for categorical variables. Statistical significance was assessed using one-way analysis of variance (ANOVA) for continuous variables and chi-square tests for categorical variables. OGTT: oral glucose tolerance test

Variable	Group A (18-25)	Group B (26-35)	Group C (36-45)	Test Statistic	P-value
Antithrombin (%)	95.2 ± 16.8	99.1 ± 17.3	102.4 ± 18.1	F(2, 447) = 3.42	0.032
OGTT (mg/dL)	175.6 ± 20.4	173.8 ± 21.1	173.2 ± 20.7	F(2, 447) = 0.03	0.979
BMI (kg/m^2^)	29.2 ± 6.1	29.8 ± 6.0	29.9 ± 6.2	F(2, 447) = 0.10	0.912
D-dimer (ng/mL)	855.4 ± 380.6	867.2 ± 379.1	870.3 ± 384.5	F(2, 447) = 1.49	0.226
Smoking (%)	58.0 (n=29)	55.6 (n=64)	57.5 (n=49)	χ^2^(2) = 6.92	0.032

Integrated risk overview

**Figure 6:**
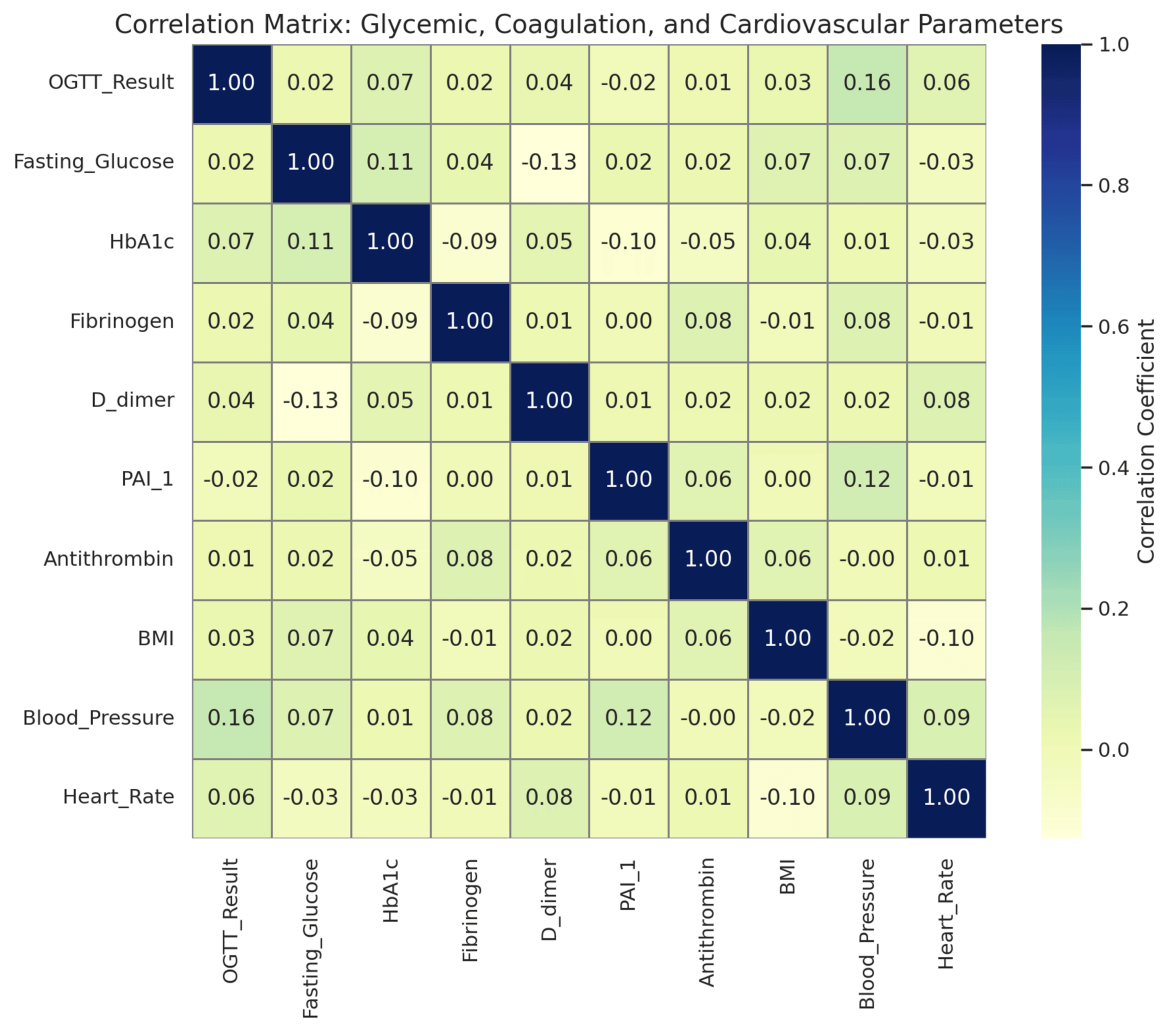
Correlation matrix of glycemic, coagulation, and cardiovascular parameters in women with gestational diabetes mellitus (GDM). OGTT: oral glucose tolerance test; PAI-1: plasminogen activator inhibitor-1; HbA1c: glycated hemoglobin

This study presents a multidimensional profile of cardiovascular and thrombotic risk among women with GDM by evaluating clinical, metabolic, hemostatic, and behavioral factors. Although no direct cardiovascular events were assessed due to the cross-sectional design, inferred risk was quantified through surrogate parameters, including hypertension, obesity, hemostatic biomarker elevation, poor glycemic control, and lifestyle factors such as smoking and physical inactivity. A detailed correlation matrix was developed to explore associations between glycemic, coagulation, and cardiovascular variables (see Figure 6). The matrix reveals generally weak correlations across most parameters. For example, OGTT results showed weak positive correlations with blood pressure (r = 0.16) and heart rate (r = 0.06), suggesting minimal direct cardiovascular impact in this dataset. Fibrinogen and D-dimer, despite their known thrombotic relevance, exhibited near-zero correlations with both glucose indices and BMI. PAI-1 showed a weak positive correlation with blood pressure (r = 0.12), possibly linking vascular strain to fibrinolytic inhibition. None of the correlations exceeded r = 0.2, and most hovered near zero, underscoring the need for multivariable models to assess complex interactions.

To address this, multivariable linear regression was conducted to evaluate the independent associations between glycemic control (fasting glucose, OGTT, HbA1c), behavioral risk (smoking, physical activity), and hemostatic markers. After adjustment for age and BMI, PAI-1 and fibrinogen remained significantly associated with fasting glucose (p < 0.01), whereas antithrombin was lower in younger participants but not significantly tied to glucose parameters. Treatment modality (e.g., insulin vs. diet) was not independently associated with changes in D-dimer, PAI-1, or fibrinogen levels. The study supports the presence of a persistent prothrombotic state, as both fibrinogen (mean 3.48 g/L) and D-dimer (mean 863 ng/mL) fell at the upper edge of third-trimester reference ranges (2.5-6.0 g/L and 500-1000 ng/mL, respectively). The elevation of PAI-1 further supports a tendency toward coagulation cascade imbalance, even though mean values remained within gestational physiological norms. These findings suggest subclinical risk rather than overt coagulopathy.

Antithrombin variability across age groups was interpreted as a potential surrogate for endothelial dysfunction, given its inverse trend with age (lowest in women aged 18-25). While no endothelial-specific biomarkers (e.g., vascular cell adhesion molecule-1 (VCAM-1)) were measured, this trend aligns with known associations between insulin resistance and endothelial impairment. Subgroup analysis revealed that smokers had modestly elevated PAI-1 and D-dimer levels compared to non-smokers (p < 0.05), strengthening the link between behavior and coagulation risk. Genetic predisposition was inferred through family history of CVD and diabetes, as no genetic screening was conducted. This variable was treated as binary (yes/no) and not weighted for degree of relation or early onset.

Although no validated cardiovascular risk calculator exists for pregnancy-specific populations, the study highlights a gap that warrants urgent attention. In the meantime, incorporating a modified scoring model and integrating basic biomarker panels with behavioral screening may offer a practical risk assessment tool. For implementation in resource-limited settings such as Pakistan, the study advocates for multidisciplinary antenatal care models. These should combine obstetric, endocrinologic, and hematologic expertise, supported by community health outreach and digital risk tracking systems to optimize care continuity and intervention timing.

## Discussion

This study sheds light on the complex interplay between metabolic, hemodynamic, and hemostatic parameters among 250 women diagnosed with GDM, revealing a multifactorial risk profile for cardiovascular and thrombotic complications. The elevated glycemic indices, OGTT (mean = 174.2 mg/dL, n = 250), fasting glucose (mean = 91.3 mg/dL, n = 250), and HbA1c (mean = 6.41%, n = 250), reflect persistent hyperglycemia, a core contributor to endothelial dysfunction and vascular damage [15]. This study aimed to characterize the cardiovascular and hemostatic profile of women with GDM and explore associations between glycemic control, treatment modalities, and thrombotic risk markers. A noteworthy observation was the substantial proportion of participants receiving antiplatelet (70.4%) and statin therapy (64.8%). While this appears to deviate from standard obstetric guidelines, which generally advise caution or contraindicate statins and dual antiplatelets in pregnancy, these medications were typically prescribed prior to conception or initiated under specialist supervision due to high pre-existing cardiovascular risk [15,16]. Their continued use was likely influenced by individualized risk-benefit assessments in a tertiary care setting. Nonetheless, the assertion that such therapies were “common” should be interpreted cautiously, as their presence in this cohort may reflect referral bias toward a high-risk population.

In assessing the impact of these therapies on clinical and biochemical outcomes, subgroup analysis did not reveal statistically significant differences in coagulation markers (fibrinogen, D-dimer, PAI-1) or glycemic indices (HbA1c, fasting glucose) across treatment groups (e.g., insulin vs. metformin, or antiplatelet vs. none). This may be due to limited statistical power or treatment heterogeneity, as many participants were exposed to overlapping regimens. Importantly, no interaction terms reached significance in multivariable models, suggesting that treatment modality alone was not a primary determinant of the observed prothrombotic state [17].

The physiological influence of gestational age on hemostatic markers is well-established, with a gradual increase in procoagulant factors such as fibrinogen and D-dimer across trimesters [18]. Although all participants were enrolled in the third trimester, gestational week was not controlled for in the statistical models, representing a limitation. Variability in gestational age (weeks 28-40) may have influenced marker concentrations and should be accounted for in future studies through stratified or adjusted analyses [19]. An age-related trend in antithrombin levels was observed, with significantly lower levels in younger women (18-25 years) compared to those aged 36-45. This is counterintuitive, as older age typically correlates with greater vascular risk. One possible explanation is that younger women with GDM may have more pronounced insulin resistance, metabolic inflammation, or unrecognized prothrombotic tendencies - mechanisms that may contribute to antithrombin depletion [20]. Alternatively, nutritional deficiencies or genetic variants affecting antithrombin synthesis could play a role in this demographic and merit further investigation.

In comparing our findings with existing literature, mean fibrinogen (3.48 g/L) and D-dimer (863 ng/mL) levels in this cohort were within, but toward the higher end of, third-trimester reference ranges. These values are consistent with reports from other GDM studies [21] but may indicate an exaggerated prothrombotic response in our population, possibly due to compounding metabolic, behavioral, or treatment-related factors. This underscores the clinical significance of subclinical coagulation abnormalities in GDM, even in the absence of overt thrombosis. The presence of a persistent prothrombotic state, characterized by elevated fibrinogen, D-dimer, and PAI-1 in a substantial proportion of participants, suggests the need for more nuanced clinical screening protocols. While universal thromboprophylaxis is not indicated in GDM, selective monitoring of coagulation markers may help identify high-risk subgroups. We recommend that future studies evaluate the predictive value of fibrinogen, D-dimer, PAI-1, and antithrombin for vascular complications postpartum or in subsequent pregnancies [19].

Treatment heterogeneity, particularly the overlap of insulin, metformin, and statin use, poses a risk of confounding the association between metabolic dysregulation and hemostatic imbalance. Although statistical adjustments were attempted, residual confounding is likely and should be addressed in prospective studies with uniform treatment protocols. Finally, the study is limited by its retrospective design and reliance on electronic medical records, which may contain missing or incomplete data. Smoking status, physical activity, and socioeconomic factors were not always systematically recorded, and some laboratory values lacked time stamps to precisely align with gestational age. These limitations highlight the need for standardized data capture systems in maternal health research, especially in resource-limited settings.

## Conclusions

This study demonstrated a significant association between GDM and increased thrombotic and cardiovascular risk. Women with GDM exhibited marked disturbances in glucose metabolism and coagulation parameters, including elevated fibrinogen, D-dimer, and PAI-1 levels, along with reduced antithrombin activity. These findings suggested that GDM represented not only a transient glycemic disorder but also a broader metabolic and proinflammatory state involving endothelial dysfunction. The term “vascular strain” referred to measurable elevations in hemostatic markers and blood pressure in the absence of overt thrombosis, indicating subclinical vascular stress. Behavioral factors such as smoking and physical inactivity, along with clinical conditions including obesity, hypertension, and a family history of diabetes, further contributed to the adverse risk profile. Treatment modalities, including insulin, metformin, and lifestyle interventions, did not show significant differences in modifying hemostatic markers, underscoring the importance of individualized risk evaluation. Routine cardiovascular monitoring, such as D-dimer, fibrinogen, and antithrombin assessment, alongside validated risk scoring and blood pressure surveillance, appeared valuable in identifying high-risk individuals during pregnancy and postpartum. Continued follow-up of coagulation profiles after delivery was also warranted due to the potential persistence of a prothrombotic state. In low-resource settings, multidisciplinary approaches might have been adapted using nurse-led clinics, mobile health tools, and integrated obstetric-metabolic care models. Further prospective and interventional studies were needed to evaluate whether early detection and targeted therapies addressing hemostatic dysfunction could mitigate long-term cardiovascular risk in women with GDM.
